# Changes in tinnitus after vestibular schwannoma surgery

**DOI:** 10.1038/s41598-019-38582-y

**Published:** 2019-02-11

**Authors:** Jing-Jing Wang, Yan-Mei Feng, Hui Wang, Ya-Qin Wu, Hai-Bo Shi, Zheng-Nong Chen, Shan-Kai Yin

**Affiliations:** Otolaryngology Institute, Affiliated Sixth People’s Hospital, Shanghai Jiao Tong University, 600 Yishan Road, Shanghai, 200233 China

## Abstract

We designed a prospective study to evaluate changes in tinnitus after vestibular schwannoma (VS) surgery. Subjects included 41 patients who were diagnosed with a VS and underwent translabyrinthine microsurgery (TLM) between January 2015 and May 2016. All patients underwent related examinations and were asked to answer the Tinnitus Handicap Inventory (THI) scale and a visual analog scale (VAS) of tinnitus severity both pre- and postoperatively. Of the 41 patients, 31 (75.6%) suffered from tinnitus before surgery. Microsurgery was associated with an overall decrease in tinnitus (*p* < 0.001). There was a significant improvement in THI and VAS scores after surgery (*p* = 0.001 and *p* = 0.005, respectively). The decrease in THI scores in the low-frequency group was significantly larger than that of the mid- and high-frequency groups after surgery (*p* = 0.034 and *p* = 0.001, respectively). The loudness of tinnitus decreased significantly after surgery (*p* = 0.031). Tinnitus in patients with VS improved after TLM. Patients with mid-/high-frequency tinnitus and louder tinnitus preoperatively seemed to have a worse prognosis than those with low-frequency and quieter tinnitus.

## Introduction

Tinnitus is the perception of sound in the absence of an external sound and usually results from a disorder of the somatosensory system or the auditory system^1^. It is a frequent symptom of vestibular schwannoma (VS), occurring in more than half of all patients^2,3^. In the past, most surgeons aimed to completely remove the tumor while preserving facial nerve function and hearing; however, recently, more attention has been paid to the patient’s quality of life^2^. Because tinnitus can reduce the quality of life in these patients^4–6^, further evaluation of tinnitus in patients with VS should be considered.

Recently, several retrospective studies have been published focusing on changes in tinnitus after microsurgery; however, the results of these papers have varied. Most of these papers showed that tinnitus improved after surgery regardless of whether translabyrinthine microsurgery (TLM), a retrosigmoid approach, or a middle cranial fossa approach was used^2,3,7^. These findings indicate that tinnitus originates in peripheral organs such as the cochlea or cochlear nerve^8,9^. However, some patients still suffer from tinnitus even after tumor removal and vestibulocochlear nerve section, which supports the theory that tinnitus is likely a symptom of central origin^10–14^. The objective of this study was to evaluate changes in tinnitus after VS microsurgery.

The Tinnitus Handicap Inventory (THI) is a reliable and valid measure of tinnitus-related handicap and is a questionnaire that is internationally acknowledged^15,16^ (see Supplementary Table). Patients without tinnitus have a handicap score of 0 on the THI, and higher THI scores correspond to worse tinnitus^17,18^. As in pain research^19^, visual analog scale (VAS) scales are increasingly being used to assess treatment-induced changes to the extent of annoyance caused by tinnitus^20^. With a VAS scale, patients self-grade the severity of tinnitus with possible scores ranging from 0 to 10 points; a score of 0 indicates no symptoms and a score of 10 indicates extremely loud tinnitus that seriously affects a patient’s daily life^19^.

## Patients and Methods

### Ethical considerations

The study protocol was approved by the Institutional Review Board of the Affiliated Sixth People’s Hospital of Shanghai Jiao Tong University, and all methods were performed in accordance with the relevant guidelines and regulations. Informed consent for study participation was obtained from all participants.

### Participants, Setting, and Study Design

In total, 41 patients diagnosed with unilateral VS were enrolled in this prospective study between January 2015 and May 2016 in the Department of ENT Head and Neck Surgery at the Sixth People’s Hospital affiliated with Shanghai Jiao Tong University. These patients were treated with TLM because their preoperative hearing was already unserviceable, or the size of tumor was too large for hearing to be preserved. Patients who did not undergo surgery but were kept for observation and patients who underwent retrosigmoid or middle fossa approaches were excluded for this study. Microsurgery was performed by the same senior surgeon, and all patients underwent complete excision.

All patients underwent pure-tone audiometry, acoustic immittance measurements, otoacoustic emissions measurements, tinnitogram, temporal bone computed tomography (CT), and internal auditory canal enhanced magnetic resonance imaging (MRI). Factors including preoperative pure-tone audiometry, postoperative facial function (House-Brackmann)^21^, tumor size, symptom duration, and the frequency and loudness of pre- and postoperative tinnitus were analyzed.

Pure-tone averages (PTAs) were obtained by calculating the average thresholds at 500, 1000, and 2000 Hz^22^.

All internal auditory canal enhanced MRI examinations were performed on an Achieva 3.0 T MRI system (Philips Healthcare, Amsterdam, The Netherlands). In general, the cerebellopontine angle (CPA) along the long axis of the tumor was used as the maximum diameter, which was considered as the tumor size^23^. All measurements were assessed by a senior radiologist and were checked by a senior otolaryngologist. For the tumor size criteria, we referred to the 2012 Acoustic Neuroma Association membership survey and divided the tumors into three groups by size: ≤1.5 cm, 1.6–2.5 cm and ≥2.6 cm^24^.

Tinnitus frequency matching and tinnitus loudness matching were measured on tinnitograms^25^. The acoustic signal was selected based on the tinnitus reported by the patient, and the types of acoustic signals were a pure-tone, narrow-band noise, pulse, warble tone and white noise. Then, the appropriate type was chosen according to the patient’s description. The matching process usually adopted the tonal debugging technique^26^ to adjust the initial test sound to the volume of the tinnitus. According to the pure-tone audiometry and experience of the technician, the participants were presented with a starting stimulus and asked if their tinnitus pitch was higher or lower. After the participants provided their answers, the frequency of the stimulus was adjusted. If the patient answered high, the frequency was increased by 50%; if the patient answered low, the frequency was reduced by 50%. If the tinnitus pitch was equal to the test sound, the test was stopped, and the last frequency was recorded. The test was repeated three times, and the average was calculated. The single ear loudness balance test was usually used to match loudness. Using the selected frequency of tinnitus, we matched the loudness to the individual’s tinnitus loudness in 1 dB steps by asking the participant whether the tinnitus was softer or louder^27^. For patients with hearing loss, we used the healthy ear to match. In the matching test, tinnitus was classified by frequency: ≤250 Hz, low-frequency; 500–2000 Hz, mid-frequency; and ≥4000 Hz, high-frequency^22^.

The severity of tinnitus was evaluated using the THI and VAS scales. All enrolled patients were assessed via the THI and VAS scales pre- and postoperatively. The analysis of the THI scores used the total score rather than the subscales^17^.

Data are presented as the mean ± standard deviation or the median (interquartile range).

A postoperative follow-up was performed approximately one year after surgery; this included an interview regarding the patient’s postoperative state and changes in tinnitus and an internal auditory canal enhanced MRI. The follow-up rate was 100%.

### Statistical Methods

Data were analyzed using SPSS (version 20.0; IBM Corp., Armonk, NY). Data from survey responses were analyzed using Student’s *t*-test, Spearman’s correlation analysis, Chi-square test, Fisher’s exact test and analysis of variance (ANOVA). For all analyses, *p* < 0.05 was considered to indicate statistical significance.

## Results

**Figure 1:**
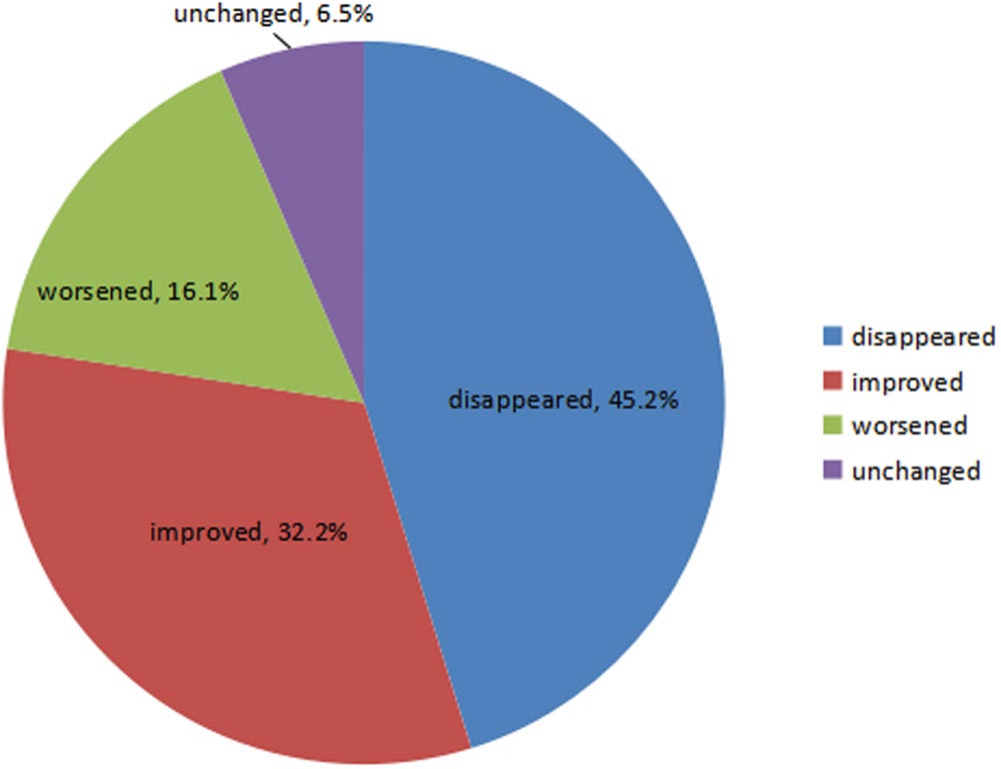
Change in tinnitus after vestibular schwannoma surgery.

There were 41 patients included in our study, and no patients had lesions in both ears (refer to Table 1 for patient characteristics). Most patients (31/41) had tinnitus before the surgery. Of these 31 patients, 14 (45.2%) reported that their tinnitus disappeared after the surgery, 10 (32.2%) reported improvement, 2 (6.5%) reported no change, and 5 (16.1%) reported worsening of tinnitus after the surgery (Fig. 1). None of the 10 patients without preoperative tinnitus had new-onset tinnitus after the surgery. Although 31 (75.6%) patients suffered from tinnitus before the operation, only 17 (41.5%) patients still had tinnitus postoperatively (including 10 patients with improved tinnitus), indicating an improvement in tinnitus incidence after microsurgery (*p* < 0.001).

**Table 1:** Characteristics of study participants.

Characteristics	Total number
Age (yrs)	51.4 ± 12.9
Gender	
Male	15 (36.6%)
Female	26 (63.4%)
Operation side	
Left	14 (34.1%)
Right	27 (65.9%)
Symptom duration (months)	12.0 (65.0)
Tumor size (cm)	1.7 (1.3)
Nature of tumor	
Cystic type	13 (31.7%)
Solid type	28 (68.3%)
Tumor size	
≤1.5 cm	13 (31.7%)
1.6–2.5 cm	15 (36.6%)
≥2.6 cm	13 (31.7%)
Preoperative pure-tone average (dB)	73.5 (36)
Preoperative tinnitus loudness (dBHL)	55 (44)
Postoperative tinnitus loudness (dBHL)	20 (74)

### THI and VAS scores

**Figure 2:**
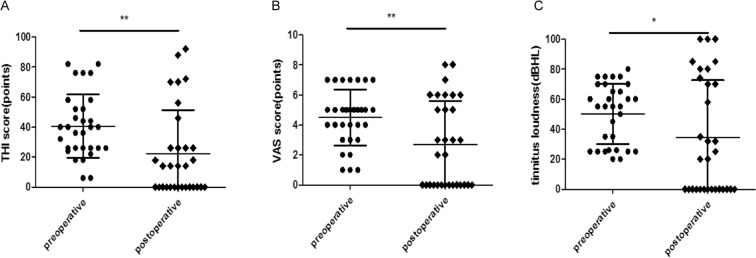
Preoperative and postoperative THI scores (**A**), VAS scores (**B**), and tinnitus loudness (**C**). *p < 0.05, **p < 0.01.

**Figure 3:**
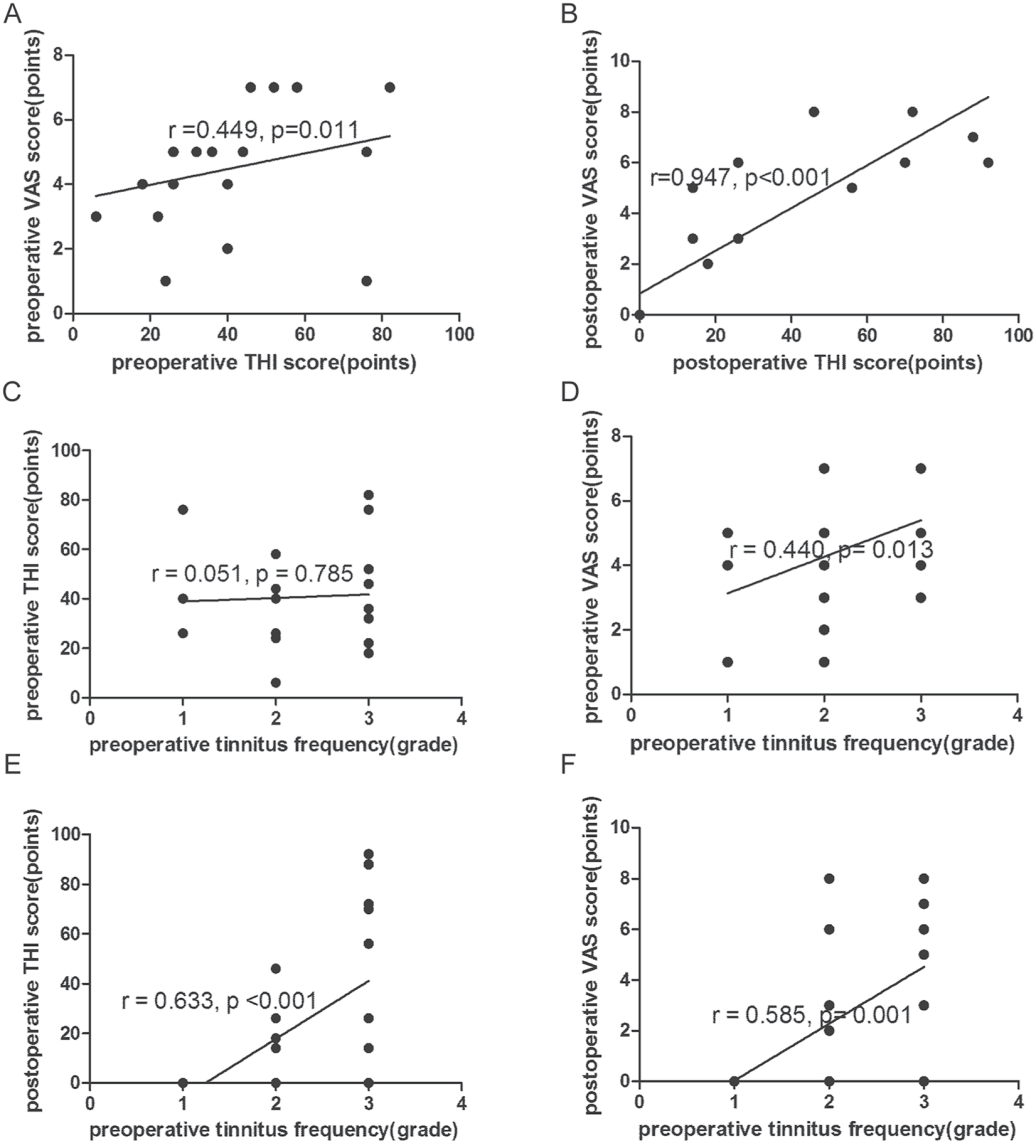
(**A**) Correlation between preoperative THI score and preoperative VAS score (r = 0.449, *p* = 0.011). (**B**) Correlation between postoperative THI score and postoperative VAS score (r = 0.947, *p* < 0.001). (**C**) Correlation between preoperative tinnitus frequency and preoperative THI score (r = 0.051, *p* = 0.785). (**D**) Correlation between preoperative frequency and preoperative VAS score (r = 0.440, *p* = 0.013). (**E**) Correlation between preoperative frequency and postoperative THI score (r = 0.633, *p* < 0.001). (**F**) Correlation between preoperative frequency and postoperative VAS score (r = 0.585, *p* = 0.001).

The overall average THI score was 40.7 ± 21.2 (mean ± standard deviation), with a range of 6–82 in the preoperative tinnitus group. The average THI score was 14 (26) (median/interquartile range), with a range of 0–92, postoperatively. This indicates a general improvement between the pre- and postoperative THI scores (*p* = 0.001, ΔTHI = −18.4 ± 29.0) (Table 2) (Fig. 2). The preoperative average VAS score was 5 (2) (range, 1–7), and the postoperative average VAS score was 2 (6) (range, 0–8). A significant improvement in the VAS score was observed after surgery (*p* = 0.005, ΔVAS = −1.8 ± 3.3) (Table 2) (Fig. 2). A moderate but significant correlation existed between the preoperative THI and preoperative VAS scores (r = 0.449, *p* = 0.011). We also found a strong correlation between the postoperative THI score and postoperative VAS score (r = 0.947, *p* < 0.001) (Fig. 3).

**Table 2:** Main results.

	Preoperative values	Postoperative values	Δd	P value
THI score	40.7 ± 21.2	14 (26)	−18.4 ± 29.0	0.001**
Low-frequency	47.3 ± 23.1	0	−47.3 ± 23.1	0.034*
Mid-frequency	32.7 ± 16.7	14 (22)	−20.2 ± 22.5	0.001**
High-frequency	46.0 ± 23.6	44.0 ± 34.2	−2.0 ± 22.5	0.077
VAS score	5 (2)	2 (6)	−1.8 ± 3.3	0.005**
Low-frequency	3.3 ± 1.9	0	−3.3 ± 1.9	0.341
Mid-frequency	4.1 ± 1.8	2 (5)	−1.8 ± 4.1	0.165
High-frequency	5 (3)	4.5 ± 2.5	−1.0 ± 2.7	0.562
Tinnitus loudness	55 (44)	20 (74)	−16.0 ± 39.4	0.031*

*p < 0.05, **p < 0.01.

In assessing the THI 20- point threshold as a significant change, we found that of these 31 patients with preoperative tinnitus, symptoms improved in 18 (58.1%), unchanged in 8 (25.8%), and worsened in 5 (16.1%) after surgery; all of these results were clinically significant (*p* < 0.001).

### Tinnitus frequency

**Figure 4:**
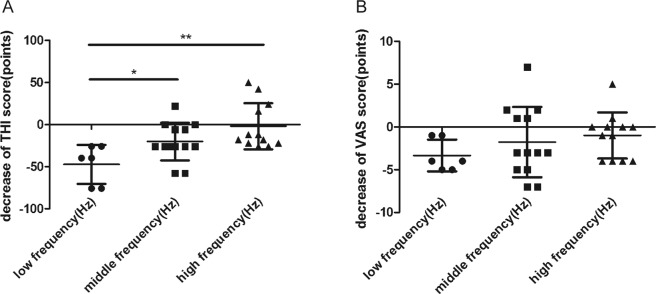
(**A**) The decrease in THI score after surgery in the low-frequency group was significantly greater than that of the mid-/high-frequency groups (ΔTHI score of low-frequency group = −47.3 ± 23.1 vs. ΔTHI score of the mid-frequency group = −20.2 ± 22.5, *p* = 0.034; ΔTHI score of the low-frequency group = −47.3 ± 23.1 vs. ΔTHI score of the high-frequency group = −2.0 ± 22.5, *p* = 0.001). (**B**) The decrease in the VAS score after the surgery did not differ among the three groups (ΔVAS score of low-frequency group = −3.3 ± 1.9 vs. ΔVAS score of the mid-frequency group = −1.8 ± 4.1 vs. ΔVAS score of the high-frequency group = −1.0 ± 2.7; *p* = 0.374).

Preoperative tinnitus was classified as low-frequency in 6 patients (19.4%), mid-frequency in 13 (41.9%), and high-frequency in 12 (38.7%). We found no significant correlation between the frequency of the preoperative tinnitus and preoperative THI (r = 0.051, *p* = 0.785) (Fig. 3). Interestingly, we found a moderate correlation between the frequency of the preoperative tinnitus and preoperative VAS score (r = 0.440, *p* = 0.013) (Fig. 3). All 6 (100%) patients with low-frequency tinnitus preoperatively had postoperative THI scores of 0. Of the 13 patients with mid-frequency tinnitus preoperatively, 10 (76.9%) patients had postoperative THI scores that decreased by an average of 28.4 points. The THI score did not change in 2 (15.4%) patients and increased by 22 points in 1 (7.7%) patient. Of the 12 patients with high-frequency tinnitus preoperatively, 8 (66.7%) had postoperative THI scores that decreased by an average of 19.5 points, and 4 (33.3%) had scores that increased by an average of 33 points. The decrease in THI score of the low-frequency group was significantly greater than that of both the mid- and high-frequency groups after surgery (ΔTHI score of low-frequency group = −47.3 ± 23.1 vs. ΔTHI score of mid-frequency group = −20.2 ± 22.5, *p* = 0.034; ΔTHI score of low-frequency group = −47.3 ± 23.1 vs. ΔTHI score of high-frequency group = −2.0 ± 22.5, *p = *0.001) (Table 2) (Fig. 4). All 6 (100%) patients with preoperative low-frequency tinnitus had postoperative VAS scores of 0. Of the 13 patients with mid-frequency tinnitus preoperatively, 8 (61.5%) patients had postoperative VAS scores that decreased by an average of 4 points, and 5 (38.5%) had scores that increased by an average of 2 points. Of the 12 patients with high-frequency tinnitus preoperatively, 6 (50.0%) had postoperative VAS scores that decreased by an average of 4 points. The VAS scores did not change in 4 (33.3%) patients and increased by 3 points in 2 (16.7%) patients. However, the decrease in the VAS score after surgery did not differ among the three groups (ΔVAS score of low-frequency group = −3.3 ± 1.9 vs. ΔVAS score of mid-frequency group = −1.8 ± 4.1 vs. ΔVAS score of high-frequency group = −1.0 ± 2.7; *p* = 0.374) (Table 2) (Fig. 4). The preoperative frequency was weakly correlated with the postoperative THI and postoperative VAS scores (r = 0.633, *p* < 0.001; r = 0.585, *p* = 0.001, respectively) (Fig. 3).

### Tinnitus loudness

**Figure 5:**
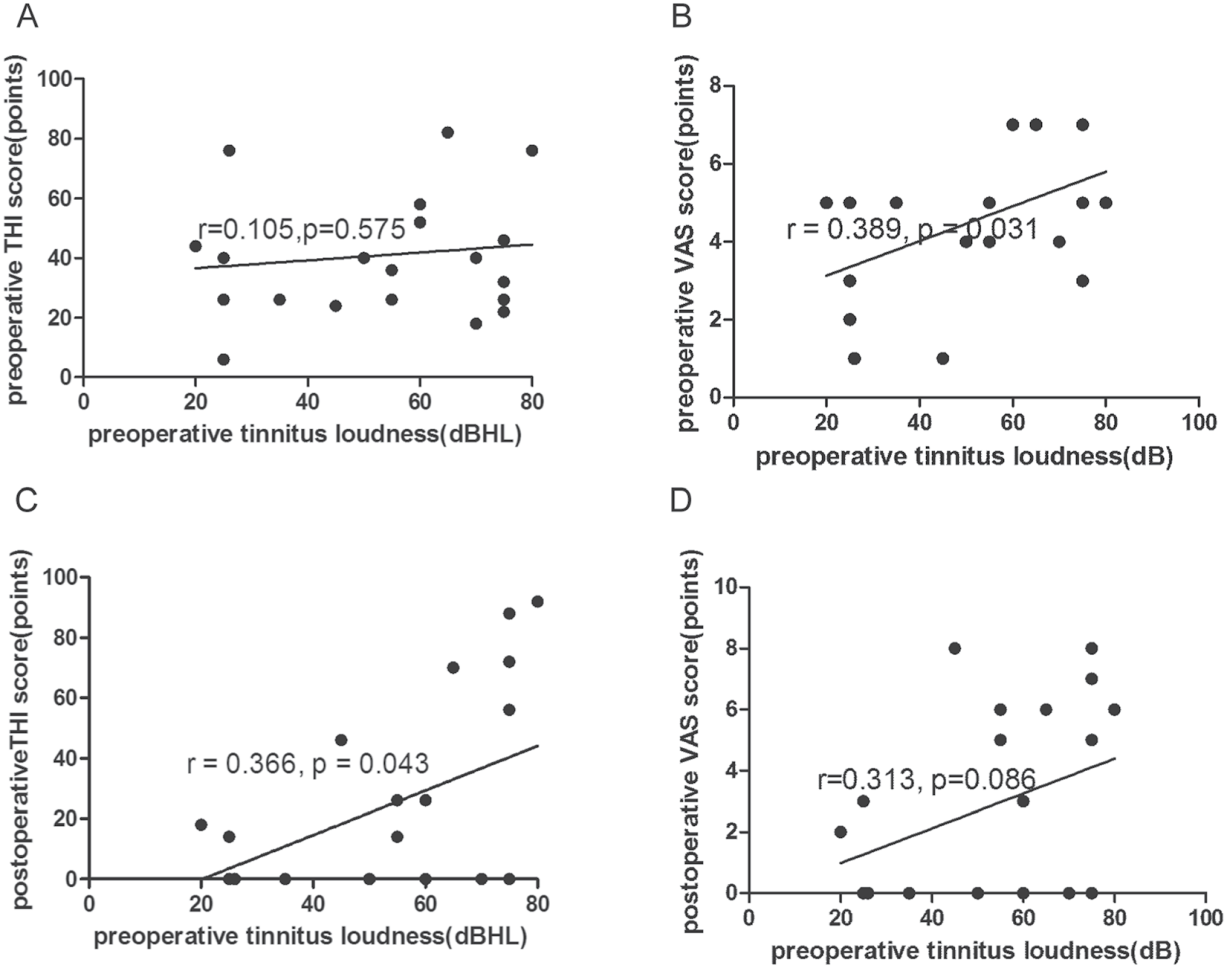
(**A**) Correlation between preoperative tinnitus loudness and preoperative THI score (r = 0.105, *p* = 0.575). (**B**) Correlation between preoperative tinnitus loudness and preoperative VAS score (r = 0.389, *p* = 0.031). (**C**) Correlation between preoperative tinnitus loudness and postoperative THI score (r = 0.366, *p* = 0.043). (**D**) Correlation between preoperative tinnitus loudness and postoperative VAS score (r = 0.313, *p* = 0.086).

The loudness of tinnitus after surgery decreased in 19 patients, increased in 10 patients, and remained unchanged in 2 patients. Among the 31 patients with preoperative tinnitus, the average loudness was 55 (44) dBHL, ranging from 20 to 80 dBHL. The postoperative tinnitus loudness ranged from 0 to 100 dBHL, with an average of 20 (74) dBHL. Thus, the loudness of tinnitus decreased significantly after surgery compared with the preoperative value (Δloudness = −16.0 ± 39.4; *p* = 0.031) (Table 2) (Fig. 2). We found no significant correlation between the loudness of preoperative tinnitus and the preoperative THI score (r = 0.105, *p* = 0.575) (Fig. 5). Interestingly, we found a weak correlation between the loudness of preoperative tinnitus and preoperative VAS score (r = 0.389, *p* = 0.031) (Fig. 5). Greater preoperative loudness was weakly correlated with a higher THI score postoperatively (r = 0.366, *p* = 0.043) (Fig. 5). However, no significant correlation was observed between preoperative loudness and the postoperative VAS score (r = 0.313, *p* = 0.086) (Fig. 5).

### Tumor size

**Figure 6:**
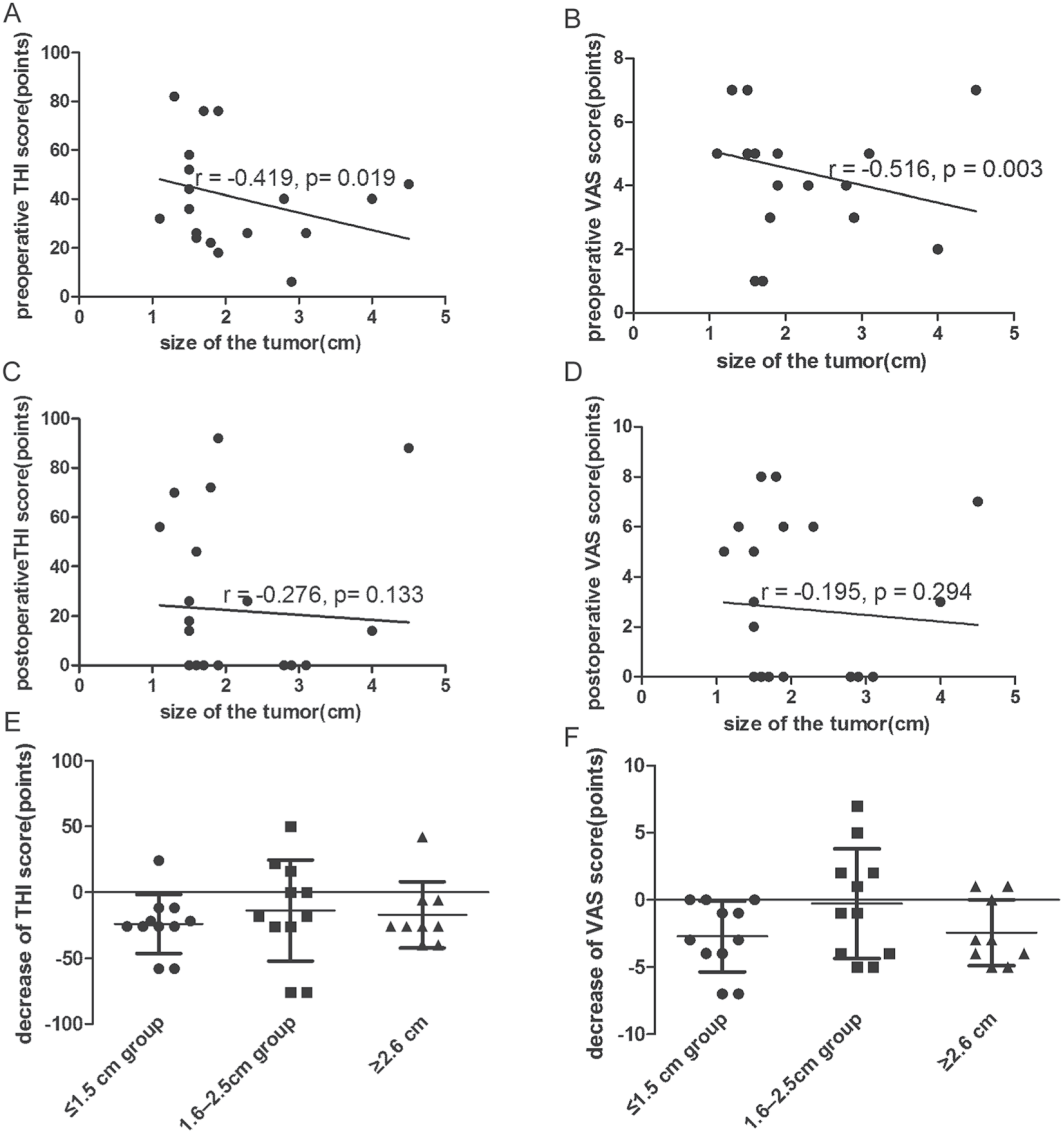
(**A**) Correlation between tumor size and preoperative THI score (r = −0.419, *p* = 0.019). (**B**) Correlation between tumor size and preoperative VAS score (r = −0.516, *p* = 0.003). (**C**) Correlation between tumor size and postoperative THI score (r = −0.276, *p* = 0.133). (**D**) Correlation between tumor size and postoperative VAS score (r = −0.195, *p* = 0.294). (**E**) The decrease in THI score did not differ among the three size groups (*p* = 0.718). (**F**) The decrease in VAS score did not differ among the three size groups (*p* = 0.165).

In the preoperative tinnitus group, the VS tumor size ranged from 1.1 to 4.5 cm initially, with a mean tumor size of 1.7 (1.3) cm. The patients were divided into three groups according to tumor size: ≤1.5 cm (*n* = 11, 35.5%); 1.6–2.5 cm (*n* = 11, 35.5%); and ≥2.6 cm (*n* = 9, 29.0%). Tumor size was correlated with the preoperative THI score and preoperative VAS score (r = −0.419, *p* = 0.019; r = −0.516, *p* = 0.003, respectively) (Fig. 6). However, tumor size was not correlated with the postoperative THI score or postoperative VAS score (r = −0.276, *p* = 0.133; r = −0.195, *p* = 0.294, respectively) (Fig. 6). Changes in the THI and VAS scores before and after surgery did not differ among the three groups (*p* = 0.718 and *p* = 0.165, respectively) (Fig. 6). In the group without preoperative tinnitus, the mean tumor size was 2.4 ± 1.2 cm, with a minimum of 0.7 cm and a maximum of 5.0 cm.

### Age

**Figure 7:**
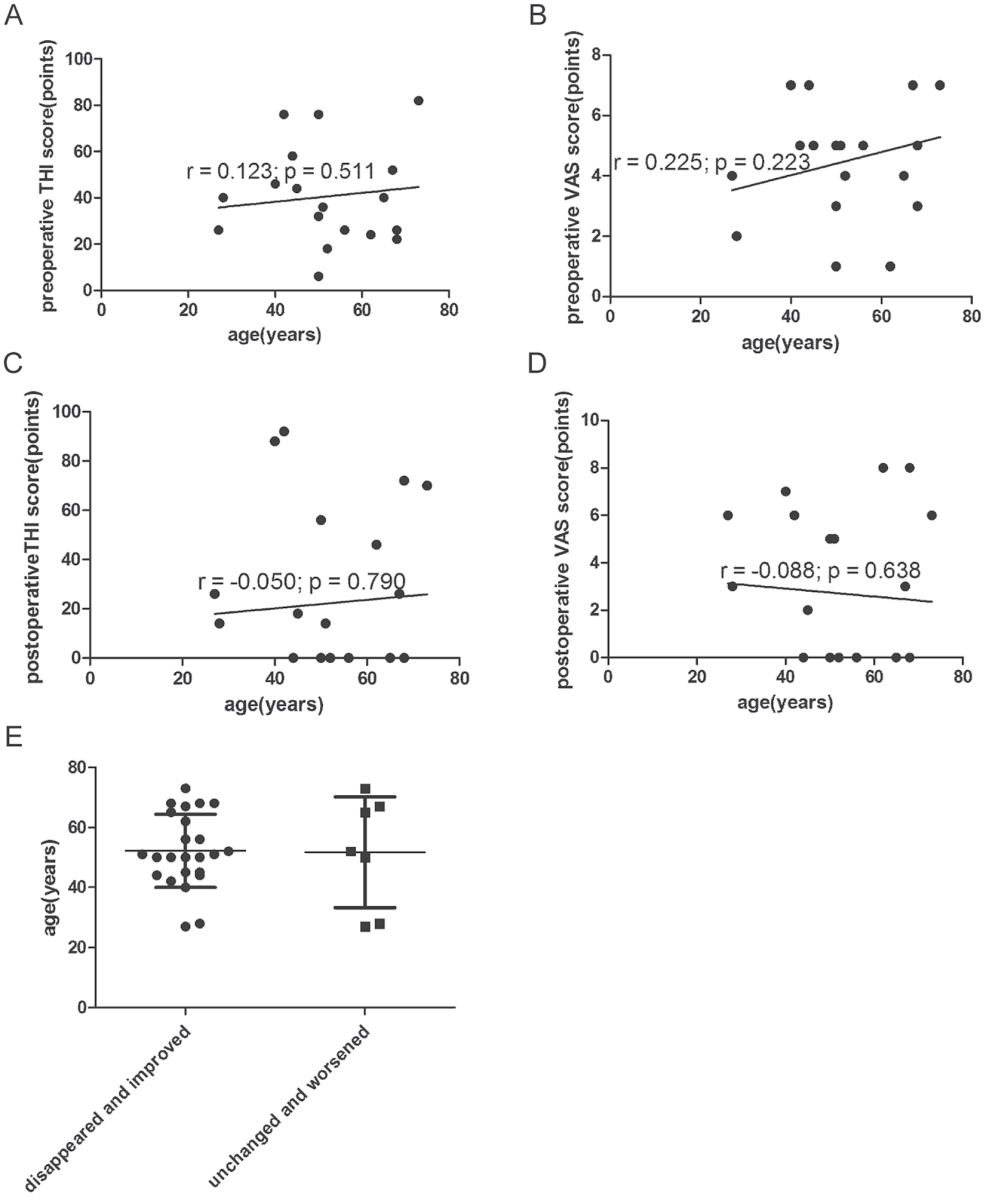
(**A**) Correlation between age and preoperative THI score (r = 0.123, *p* = 0.511). (**B**) Correlation between age and preoperative VAS score (r = 0.225; *p* = 0.223). (**C**) Correlation between age and postoperative THI score (r = −0.050, *p* = 0.790). (**D**) Correlation between age and postoperative VAS score (r = −0.088, *p* = 0.638). (**E**) The mean age of patients with disappeared and improved tinnitus versus that of patients with unchanged or worsened tinnitus (*p* = 0.939).

In 31 patients with preoperative tinnitus, the mean age of patients was 52.1 ± 13.5 years (range, 27–73 years). Age was not correlated with preoperative THI score or preoperative VAS score (r = 0.123, *p* = 0.511; r = 0.225, *p* = 0.223, respectively) (Fig. 7). Age was also not correlated with postoperative THI score or postoperative VAS score (r = −0.050, *p* = 0.790; r = −0.088, *p* = 0.638, respectively) (Fig. 7). The mean age of patients with tinnitus that disappeared or improved was 52.2 ± 12.6 years; the mean age of those with tinnitus that did not disappear or improve was 51.7 ± 18.4 years (*p* = 0.939) (Fig. 7). In 10 patients without preoperative tinnitus, the mean age was 49.8 ± 12.4 years (range, 27–73 years).

### Preoperative pure-tone audiometry

**Figure 8:**
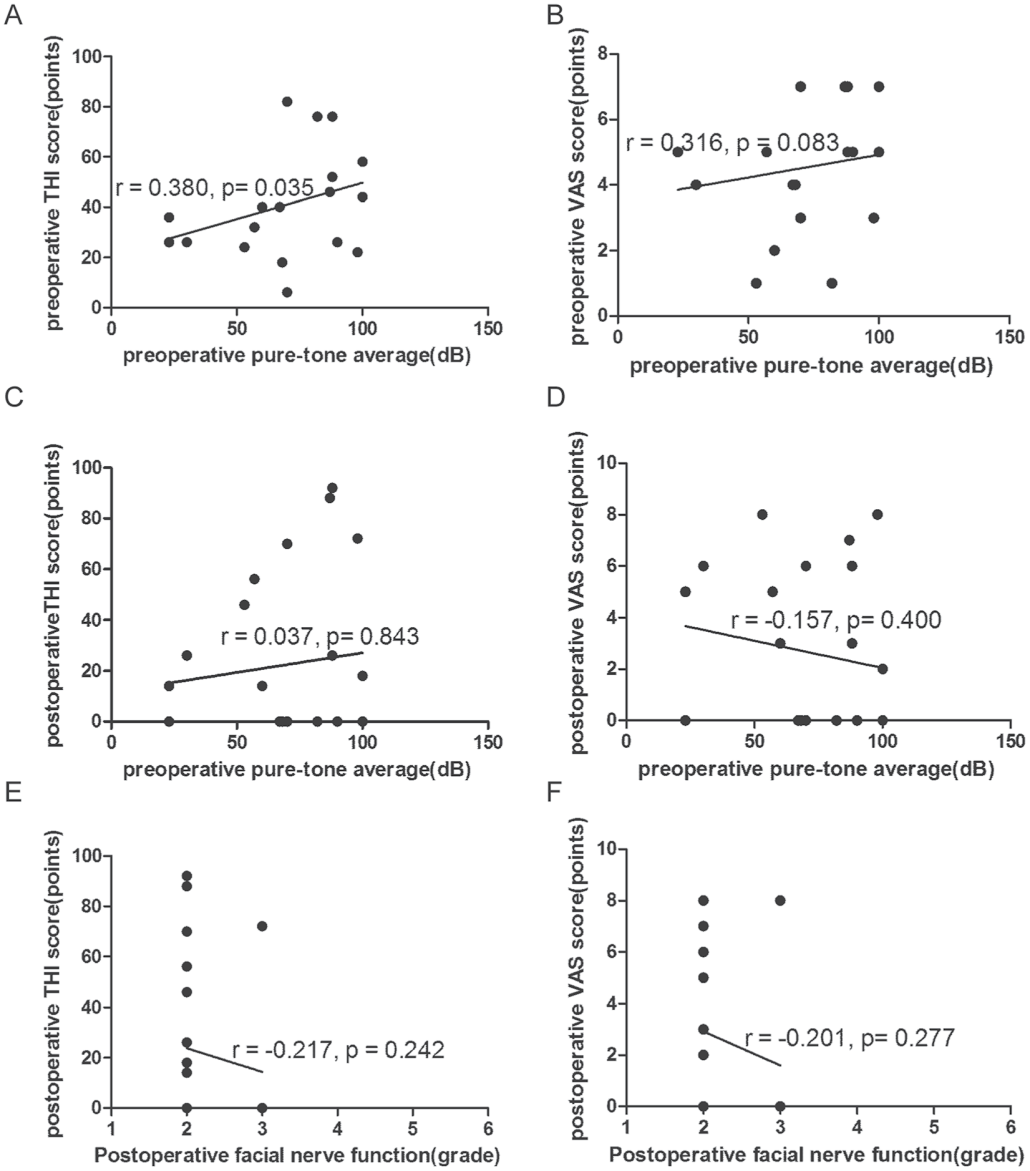
(**A**) Correlation between the preoperative pure-tone average and preoperative THI score (r = 0.380, *p* = 0.035). (**B**) Correlation between the preoperative pure-tone average and preoperative VAS score (r = 0.316, *p* = 0.083). (**C**) Correlation between the preoperative pure-tone average and postoperative THI score (r = 0.037, *p* = 0.843). (**D**) Correlation between the preoperative pure-tone average and postoperative VAS score (r = −0.157, *p* = 0.400). (**E**) Correlation between facial nerve function and postoperative THI score (r = −0.217, *p* = 0.242). (**F**) Correlation between facial nerve function and postoperative VAS score (r = −0.201, *p* = 0.277).

In 31 patients with preoperative tinnitus, the preoperative pure-tone average was 68.6 ± 25.4 dB. A weak correlation was observed between the preoperative pure-tone average and the preoperative THI score (r = 0.380, *p* = 0.035) (Fig. 8). No significant correlation was found between the preoperative pure-tone average and the preoperative VAS score (r = 0.316, *p* = 0.083) (Fig. 8). In addition, no significant correlation was observed between the preoperative pure-tone average and the postoperative THI score or postoperative VAS score (r = 0.037, *p* = 0.843; r = −0.157, *p* = 0.400, respectively) (Fig. 8). In the group without preoperative tinnitus, the preoperative pure-tone average was 98.0 (27.0) dB.

### Postoperative facial nerve function (House-Brackmann scale)

In the group with preoperative tinnitus, the House-Brackmann grade of postoperative facial nerve function was grade II in 26 (83.9%) cases and grade III in 5 (16.1%) cases; no correlation was observed between facial nerve function and the postoperative THI score or postoperative VAS score (r−0.217, *p* = 0.242; r = −0.201, *p* = 0.277, respectively) (Fig. 8). In the group without preoperative tinnitus, the postoperative House-Brackmann grade was II in 7 (70%) cases, III in 1 (10%) case, and VI in 2 (20%) cases; the postoperative THI score was 0 in every patient of this group.

### Symptom duration

**Figure 9:**
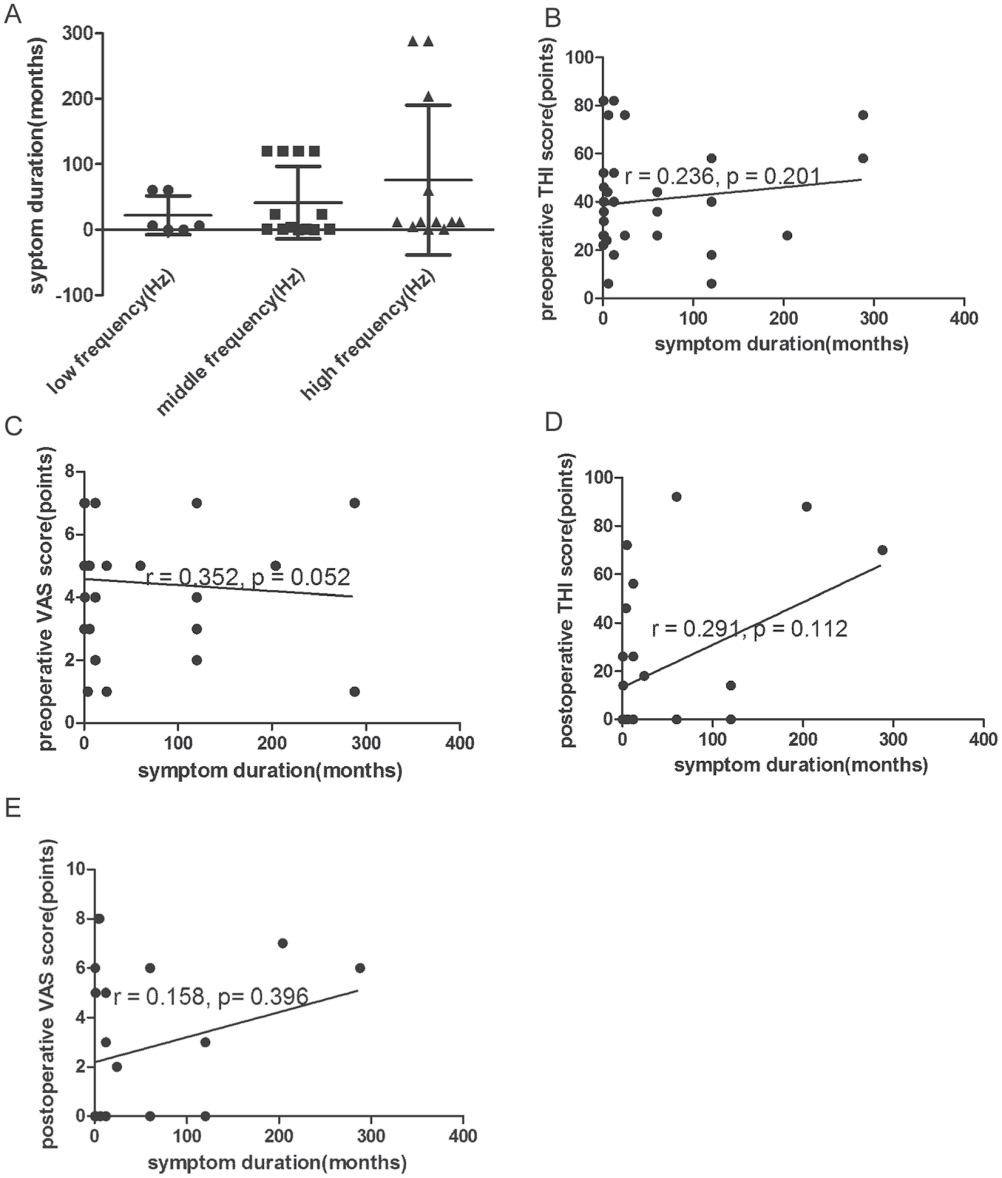
(**A**) The average symptom duration did not differ among the three frequency groups (*p* = 0.165). (**B**) Correlation between symptom duration and preoperative THI score (r = 0.236, *p* = 0.201). (**C**) Correlation between symptom duration and preoperative VAS score (r = 0.352, *p* = 0.052). (**D**) Correlation between symptom duration and postoperative THI score (r = 0.291, *p* = 0.112). (**E**) Correlation between symptom duration and postoperative VAS score (r = 0.158, *p* = 0.396).

In the preoperative tinnitus group, the average symptom duration was 12 (59) months. The average symptom duration was 6 (60) months for patients with low-frequency tinnitus, 4 (119) months for patients with mid-frequency, and 12 (161) months for patients with high-frequency tinnitus. No significant differences in symptom duration were observed between the low-/mid-frequency and high-frequency groups (*p* = 0.372) (Fig. 9). No significant correlation was observed between symptom duration and preoperative THI score or preoperative VAS score(r = 0.236, *p* = 0.201; r = 0.352, *p* = 0.052, respectively) (Fig. 9). Additionally, no significant correlation was observed between symptom duration and postoperative THI score or postoperative VAS score (r = 0.291, *p* = 0.112; r = 0.158, *p* = 0.396, respectively) (Fig. 9). In the group without preoperative tinnitus, the average symptom duration was 18 (79) months.

## Discussion

In this study, tinnitus disappeared (45.2%) or improved (32.2%) for most patients after surgery, indicating a significant overall decrease in tinnitus via microsurgery. Patients with low-frequency and quieter tinnitus preoperatively seemed to have a better postoperative prognosis than those with mid-/high-frequency or louder tinnitus before the surgery.

Previous studies have demonstrated mixed surgical results regarding its effect on tinnitus. Baguley *et al*.^17^ reported that tinnitus was neither exacerbated nor relieved after TLM, while many others found that microsurgery improved tinnitus^2,7,28–30^, consistent with our observations. The underlying mechanisms are still unclear; however, several hypotheses have been proposed. First, the TLM surgical approach sacrifices inner ear and cochlear nerve function^3^, disrupting the central auditory conduction path. Second, cochlear or auditory nerve lesions can cause increases in the discharge rates of inner hair cells and nerve fibers, and the central auditory system cannot distinguish between pathological and normal physiological excitation and produces errors in hearing, resulting in tinnitus^2,11^. From this point of view, surgery may relieve the mechanical stimulation of the auditory nerve by the tumor, thus preventing the abnormal impulses and, consequently, eliminating tinnitus.

Currently, no consensus exists on whether tinnitus relief is related to cochlear nerve dissection. Kameda *et al*.^3^ reported that tinnitus disappeared or improved in most cases after the retrosigmoid approach and showed no difference in tinnitus incidence whether the vestibulocochlear nerve was resected or not. Park *et al*.^2^ suggested that cochlear nerve section may be beneficial for the improvement of postoperative tinnitus by comparing changes in tinnitus after TLM and gamma knife radiosurgery. In this study, the chosen surgery was the translabyrinthine approach because hearing preservation was not intended. During surgery, the cochlea nerve section was definite in all cases^31^ as suggested by Park *et al*.^2^, which may be the reason for the high rates of improvement in tinnitus. Although sectioning of the cochlear nerve creates a condition unsuitable for cochlear implantation^32^, either simultaneously or prospectively, a BAHA implant is a hearing solution for these patients^33^.

We found that the postoperative prognosis was better in patients with low-frequency tinnitus than in those with mid-/high-frequency tinnitus. However, the specific reasons and mechanisms for this finding remain unclear. Interestingly, a previous study found that among patients with sudden deafness accompanied by tinnitus, the rate of recovery was better for those with low-frequency tinnitus than those with high-frequency tinnitus^34^. The author suggested that high-frequency tinnitus may be related to a longer duration and more severe injury and is therefore associated with greater difficulty in recovery. However, we found no significant relationship between the duration of tinnitus and the different frequencies.

The preoperative tinnitus loudness was significantly correlated with the postoperative tinnitus loudness in our cohort, i.e., greater loudness was correlated with greater difficulty in recovery. In theory, louder tinnitus has a greater impact on patients and therefore results in higher scores on the THI and VAS^18,19^.

Based on the report by Newman *et al*.^35^, a 20- point or larger change is considered clinically significant at the 5% confidence level^16,17^. In this study, we found statistically significant changes in the THI score, considering any change in the THI to represent a change in tinnitus. Moreover, when we reassessed our results only considering a 20- point or larger change in the THI score, these changes were also statistically significant. Therefore, VS removal caused clinically relevant changes in our cohort.

Whether the severity of tinnitus is associated with tumor size, age, preoperative pure-tone audiometry or postoperative facial nerve function remains controversial^2,4–7,16,31,36,37^. In this study, we found no correlation between them. Kohno *et al*.^31^ found that tinnitus appeared in one-fifth of the patients without preoperative tinnitus. We evaluated patients without preoperative tinnitus and found no new-onset tinnitus postoperatively.

Some published findings are either retrospective studies or demonstrate that tinnitus improved after microsurgery regardless of which approach was used^2,3,7,17^. However, our study was a prospective study and focused exclusively on the translabyrinthine route. Baguley *et al*.^17^ and Alvarez *et al*.^16^ also focused on the translabyrinthine approach. They demonstrated a change in tinnitus only by the THI scale, whereas our study is the first clinical study to reveal correlations between the preoperative THI score, VAS score, loudness and changes in tinnitus postoperatively. These tests are not interchangeable. In addition, because this study had a relatively limited sample size of 41 patients, it remains possible that unperceived differences existed between the groups^38^.

## Conclusion

Tinnitus in patients with VS decreased after TLM surgery, as measured and cross-validated by three separate measures: THI, VAS, and tinnitus loudness matching. Patients with low-frequency and quieter tinnitus preoperatively seemed to have a better postoperative prognosis than those with mid-/high-frequency or louder tinnitus before the surgery.

## Supplementary information

Supplementary Table
