# Supplementary material for: Changes in tinnitus after vestibular schwannoma surgery
Source: Sci Rep. 2019 Feb 11;9:1743. doi: 10.1038/s41598-019-38582-y (PMC6370768; doi:10.1038/s41598-019-38582-y)
Supplement: Supplementary file 1 — Supplementary Table [file 41598_2019_38582_MOESM1_ESM.pdf]

Changes in tinnitus after vestibular schwannoma surgery

Jing-Jing Wang[1#], Yan-Mei Feng[1#], Hui Wang[1], Ya-Qin Wu[1], Hai-Bo Shi[1], Zheng-Nong Chen[1*], Shan-Kai Yin[1*]

[1]Otolaryngology Institute, Affiliated Sixth People's Hospital, Shanghai Jiao Tong University, 600 Yishan Road, Shanghai 200233, China.

***Corresponding author:** Zheng-Nong Chen and Shan-Kai Yin

Otolaryngology Institute, Affiliated Sixth People's Hospital, Shanghai Jiao Tong University, 600 Yishan Road, Shanghai 200233, China. Email: jassey@126.com. Fax number: +86-2164834143. Tel: +86-2164834143

Otolaryngology Institute, Affiliated Sixth People's Hospital, Shanghai Jiao Tong University, 600 Yishan Road, Shanghai 200233, China.

Email:yinshankai@china.com. Fax number: +86-2164834143. Tel: +86-2164834143

[#]These authors have contributed equally to this work.

Acknowledgments: This study received support from the National Natural Science Foundation of China (grant No. 81371085; 81400466), Shanghai Pujiang Program (grant No. 15PJD030), and Shanghai Municipal Education Commission-Gaofeng Clinical Medicine Grant Support (grant No. 20152525 and No. 20152526).

Conflict of Interest: None declared.

Author Contributions: J.J.W., Z.N.C. and S.K.Y. designed the experiments. Y.M.F. and H.W. analysed the data. J.J.W. and Z.N.C wrote the main article. Y.Q.W., H.B.S. and Z.N.C. revised the manuscript. All authors have approved the final copy of this manuscript.

Supplementary Table Tinnitus Handicap Inventory

1. Because of your tinnitus, is it difficult for you to concentrate?	Yes	Sometimes	No
2. Does the loudness of your tinnitus make it difficult for you to hear people?	Yes	Sometimes	No
3. Does your tinnitus make you angry?	Yes	Sometimes	No
4. Does your tinnitus make you feel confused?	Yes	Sometimes	No
5. Because of your tinnitus, do you feel desperate?	Yes	Sometimes	No
6. Do you complain a great deal about your tinnitus?	Yes	Sometimes	No
7. Because of your tinnitus, do you have trouble falling to sleep at night?	Yes	Sometimes	No
8. Do you feel as though you cannot escape your tinnitus?	Yes	Sometimes	No
9. Does your tinnitus interfere with your ability to enjoy your social activities (such as going out to dinner, to the movies)?	Yes	Sometimes	No
10. Because of your tinnitus, do you feel frustrated?	Yes	Sometimes	No
11. Because of your tinnitus, do you feel that you have a terrible disease?	Yes	Sometimes	No
12. Does your tinnitus make it difficult for you to enjoy life?	Yes	Sometimes	No
13. Does your tinnitus interfere with your job or household responsibilities?	Yes	Sometimes	No
14. Because of your tinnitus, do you find that you are often irritable?	Yes	Sometimes	No
15. Because of your tinnitus, is it difficult for you to read?	Yes	Sometimes	No
16. Does your tinnitus make you upset?	Yes	Sometimes	No
17. Do you feel that your tinnitus problem has placed stress on your relationships with members of your family and friends?	Yes	Sometimes	No

18. Do you find it difficult to focus your attention away from your tinnitus and on other things?	Yes	Sometimes	No
19. Do you feel that you have no control over your tinnitus?	Yes	Sometimes	No
20. Because of your tinnitus, do you often feel tired?	Yes	Sometimes	No
21. Because of your tinnitus, do you feel depressed?	Yes	Sometimes	No
22 Does your tinnitus make you feel anxious?	Yes	Sometimes	No
23. Do you feel that you can no longer cope with your tinnitus?	Yes	Sometimes	No
24. Does your tinnitus get worse when you are under stress?	Yes	Sometimes	No
25. Does your tinnitus make you feel insecure?	Yes	Sometimes	No
Its 25 questions asks patients about difficulties that may be related to their tinnitus, to which patients may respond yes (4 points), sometimes (2 points), or no (0 points).			
